# Caffeine in cerebrovascular research: To withdraw or not to withdraw?

**DOI:** 10.1113/EP092703

**Published:** 2025-06-05

**Authors:** Brooke R. Shepley, Kevin J. Milne, Anthony Richard Bain

**Affiliations:** ^1^ Department of Kinesiology University of Windsor Ontario Canada

## 1 INTRODUCTION

Caffeine is among the most widely used substances, with ∼80% of the global population consuming an average of 200 mg/day (Samoggia & Rezzaghi, 2021). Following consumption (e.g., through coffee or tea), caffeine reaches maximal plasma concentrations within 30–120 min and has a half‐life of 4–6 h (Fredholm et al., 1999). The physiological effects of caffeine are largely attributed to its action as an adenosine receptor antagonist, particularly on the A_1_ and A_2_ subtypes (Ribeiro & Sebastião, 2010). In this respect, adenosine is a multifaceted neuromodulator, in addition to a nucleoside with potent vasoactive properties, depending on the target receptor and organ (Benarroch, 2009). Of particular importance in the field of cerebrovascular physiology is to consider: (1) the direct cerebrovascular modulatory effects of caffeine; (2) the indirect cerebrovascular effects (cerebral perfusion pressure and ventilation) of caffeine; and (3) the effects of withdrawal. We argue that this last component is often ignored in research settings, because most studies adhere to a 6–24 h withdrawal period. Indeed, controlling for caffeine is complicated by its ubiquity in use, which begs the question of what the ‘true’ cerebral blood flow (CBF) baseline response is. The physiological impacts of withdrawal must be considered, and it is essential to evaluate whether the normal consumption or, in most cases, abnormal abstention of caffeine in research best reflects lived physiology.

## 2 MECHANISM OF ACTION OF CAFFEINE

Caffeine is a non‐selective antagonist of adenosine and interacts with cerebral A_1_, A_2A_ and A_2B_ receptors, leading to neural activation in the A_1_ subtype and vascular activation in the A_2_ subtypes (Addicott et al., 2009; Dodd et al., 2015). Importantly, the A_2A_ and A_2B_ receptors are present in the smooth muscle of the cerebrovasculature, with the A_2A_ subtype located predominantly in pial arteries and arterioles, whereas the A_2B_ receptors are situated primarily on parenchymal vessels (Addicott et al., 2009; Pelligrino et al., 2010). In short, when caffeine binds to A_2_ subtypes, adenosine‐induced vasodilatation (Addicott et al., 2009) is inhibited, leading to increased cerebrovascular tone (Dodd et al., 2015).

Adenosine receptors (primarily A_2A_) are also abundant in the peripheral vasculature. Caffeine‐mediated inhibition of these receptors results in an increase in systemic vascular resistance and blood pressure (Pincomb et al., 1988; Umemura et al., 2006). In addition to the antagonistic effects of caffeine, it also stimulates the CNS, thereby increasing circulating catecholamines, such as noradrenaline and adrenaline, which can increase heart rate, myocardial contractility and alveolar ventilation (Papadelis et al., 2003; Pincomb et al., 1988).

## 3 RESTING CEREBRAL BLOOD FLOW AND CAFFEINE

Conceptually, caffeine can alter CBF by: (1) cerebrovascular constriction via inhibition of adenosine‐mediated dilatation; (2) increased cerebral perfusion pressure via increased arterial blood pressure; and (3) increased cerebrovascular resistance if an increase in respiration lowers the arterial partial pressure of CO_2_ ($P_{\mathrm{aC}O_{2}}$). En masse, the direct cerebrovascular effects of adenosine inhibition combined with a small reduction in $P_{\mathrm{aC}O_{2}}$ appear to outweigh the influence of increased cerebral perfusion pressure. For example, acute caffeine consumption of 250 mg reduces resting CBF by 20%–30% (Addicott et al., 2009; Turnbull et al., 2017). Importantly, the magnitude of the decline is contingent upon the amount of caffeine regularly consumed; i.e., inexperienced or low (e.g., 45 mg/day) users exhibit heightened sensitivity to the effects of caffeine (Addicott et al., 2009; Turnbull et al., 2017). Chronic caffeine exposure leads to the development of tolerance and a reduction in cardiovascular perturbations (Addicott et al., 2009; Turnbull et al., 2017). Indeed, participants who were administered caffeine equivalent to their usual daily intake (an average of 333 mg/day), with half the dose administered in the morning and the other half in the afternoon, did not exhibit any significant differences in CBF when compared with baseline measurements after the afternoon dose (Jones et al., 2000). Likewise, caffeine consumption of 150 mg resulted in a decrease in CBF 30 min after consumption; however, it returned to values comparable to ‘baseline’ 2 h after intake (Couturier et al., 1997). Furthermore, although naive users exhibit considerable decreases in CBF after 75 mg of caffeine, this dose had no impact on habitual users (Kennedy & Haskell, 2011).

The reduction in CBF with caffeine consumption appears to be mediated primarily by the direct vasoconstricting effects of inhibiting adenosine reception and only secondarily from the increase in minute ventilation. For example, an acute dose of 250 mg of caffeine has been found to increase minute ventilation by ∼6% (Richmond, 1949), which would correspond to a reduction in partial pressure of arterial carbon dioxide ($P_{\mathrm{aC}O_{2}}$) by slightly <6% (where PaCO2=V˙CO2/V˙CO2V˙AV˙A (rate of alveolar ventilation), i.e., the alveolar ventilation equation), but only if assuming that rate of carbon dioxide production (${\dot{V}}_{CO_{2}}$) remains constant. Yet, caffeine increases the ${\dot{V}}_{CO_{2}}$ (Powers et al., 1986). Ultimately, there are limited data where end‐tidal partial pressure of CO_2_ (or better, $P_{\mathrm{aC}O_{2}}$) is measured after caffeine ingestion (and breathing room air), except following a large dose of 650 mg, whereby end‐tidal partial pressure of CO_2_ decreased by ∼4 mmHg (D'Urzo et al., 1990). On average, this alone would decrease the CBF by 4%–12% (Willie et al., 2014), but it should be noted that 650 mg of caffeine is a very high dose. As such, at least within normal doses, the ventilatory impacts of caffeine on CBF are likely to be minor.

The inability of the increase in cerebral perfusion pressure to offset reductions in CBF with caffeine is likely to be related to the static cerebral autoregulatory mechanisms, whereby CBF remains relatively constant with gradual changes in mean arterial pressure within ∼±10 mmHg (Brassard et al., 2021). An acute 200–250 mg dose of caffeine increases systolic pressure by only 3–14 mmHg and diastolic pressure by 4–13 mmHg (Grant et al., 2018). Importantly, similar to the direct cerebrovascular responses, the range of the pressor response is dependent on the tolerance to caffeine, whereby it is less in habitual users (Turnbull et al., 2017).

## 4 CEREBRAL BLOOD FLOW AND WITHDRAWAL

Chronic caffeine use appears to increase the number and activity of adenosine receptors (Addicott et al., 2009). Specifically, chronic consumption of caffeine leads to an enhancement in the expression of A_1A_ receptors in mice (Johansson et al., 1993; Shi & Daly, 1999), in addition to elevated plasma levels of adenosine (Ribeiro & Sebastião, 2010) and increased adenosine sensitivity (Addicott et al., 2009). Consequently, caffeine must then compete with adenosine, which has a high affinity for A_1A_ and A_2A_ receptors (Ribeiro & Sebastião, 2010), ultimately resulting in diminished effects (tolerance). Although this attribute of caffeine tolerance might reduce the physiological effects of caffeine, such as decreased vasoconstriction, the increase in adenosine receptors might also amplify the effects of adenosine during caffeine withdrawal (Addicott et al., 2009). Increased adenosine signalling occurs, owing to an increased probability of receptor–ligand interactions (i.e., caffeine‐induced increased adenosine sensitivity) that might exacerbate the physiological symptoms of withdrawal. For example, in regular caffeine users, withdrawal leads to an amplification of CBF corresponding to the severity of withdrawal headaches (Addicott et al., 2009).

For regular users, withdrawal symptoms can appear within 12–24 h and can persist for 2–9 days, resulting in reduced reaction times, decreased alertness and flu‐like symptoms (Juliano & Griffiths, 2004; Juliano et al., 2019). Withdrawal is also frequently associated with symptoms such as irritability, anxiety, insomnia, fatigue, and moderate to severe headaches (Couturier et al., 1997; Dews et al., 2002). The psychological dependence on caffeine alone can also influence physiological withdrawal symptoms. For instance, participants who received decaffeinated coffee but were informed that it contained caffeine reported fewer withdrawal symptoms in comparison to those who were told it was decaffeinated (Juliano et al., 2019). With respect to CBF, caffeine withdrawal (i.e., 24 h) has been demonstrated to increase cerebral blood velocity in the middle, basilar and posterior cerebral arteries by ∼15% and to decrease pulsatility in the middle cerebral arteries (Couturier et al., 1997; Jones et al., 2000). Habitual caffeine consumers also demonstrate a reduction in blood pressure within 12–24 h, whereby mean arterial pressure declines by ∼5–6 mmHg on average (Lane, 1997; Phillips‐Bute & Lane, 1997). Not surprisingly, the impact of caffeine withdrawal on CBF is also dependent upon the quantity that is regularly ingested, with increased consumption leading to worsened withdrawal symptoms and elevated resting CBF (Addicott et al., 2009; Dodd et al., 2015).

## 5 CEREBROVASCULAR RESEARCH IN HABITUAL CAFFEINE USERS

Given the implications of both caffeine consumption and withdrawal on CBF, the question remains: should research participants refrain from or be allowed caffeine consumption prior to experimental studies on cerebrovascular haemodynamics? Common practice is to refrain from caffeine consumption for 6–12 h prior to experimentation to capture the ‘baseline’ state of human physiology. However, this assumption does not hold true for habitual caffeine consumers. Instead, at minimum, it reflects a state of withdrawal, but it also fails to represent accurately the ‘normal physiology’ encountered in the daily life of a caffeine user.

Cerebrovascular tone is largely regulated by four interconnected factors: blood gases, metabolism, perfusion pressure and autonomic tone. Cerebrovascular reactivity, neurovascular coupling (NVC) and cerebral autoregulation (CA) testing are widely recognized assessments used to evaluate these four factors through vascular responsiveness to blood gases (CO_2_ and O_2_), neuronal activation/metabolism and perfusion pressure, respectively (Willie et al., 2014).

With respect to cerebrovascular CO_2_ reactivity, ∼150–300 mg of caffeine does not appear to influence the change in CBF (Blaha et al., 2007; Chen & Parrish, 2009b). Although adenosine has been implicated as one of many tenable signalling molecules for cerebral vasomotor changes in response to alterations in $P_{\mathrm{aC}O_{2}}$ (Hoiland et al., 2019), these data indicate that it is likely to be of lesser importance than other known factors, including nitric oxide and arachidonic acid metabolites. Likewise, and perhaps even more surprisingly, theophylline, a potent adenosine antagonist with properties similar to caffeine, does not impact the vasomotor response to hypoxaemia (Bowton et al., 1988; Hoiland et al., 2017). In turn, these findings suggest that caffeine consumption might not be a major concern in studies where cerebrovascular reactivity is a primary outcome. However, the complete cessation of caffeine consumption for 3 months has been shown to improve cerebrovascular reactivity to CO_2_ (Gil et al., 2022), at least in participants suffering from migraines.

Regarding NVC, the process by which increased neuronal activity triggers localized increase in CBF, caffeine causes a neurovascular ‘uncoupling’ effect (Chen & Parrish, 2009b; Dodd et al., 2015). Collectively, despite reductions in CBF, whole‐brain cerebral metabolism is fully compensated for via an increase in the fraction of oxygen extracted (Xu et al., 2015). Yet, caffeine withdrawal can be equally problematic, particularly in chronic users. The elevated adenosine sensitivity that comes with chronic caffeine use might alter NVC during caffeine withdrawal because heightened adenosine signalling could enhance CBF through vasodilatation (Zhu et al., 2022). Additionally, adenosine is thought to inhibit glutamate release in presynaptic neurons, which is essential to the NVC response (Nam et al., 2012). Consequently, an increase in adenosine signalling might enhance these vasodilatory effects while simultaneously inhibiting glutamate release, resulting in a discrepancy between CBF and neuronal activity (Nam et al., 2012).

Lastly, there are no studies directly examining the effects of caffeine on CA. However, the known effects of caffeine to increase mean arterial pressure in a dose‐dependent manner (Mesas et al., 2011) or of caffeine withdrawal to reduce mean arterial pressure must be considered. Moreover, the stimulatory effects of caffeine on the sympathetic nervous system might alter the dynamic elements of CA within the autoregulatory zone (Brassard et al., 2021; Hamner & Tan, 2014).

## 6 RECOMMENDATIONS

Attempts to control the cardiovascular effects of caffeine have become common practice, but the protocols vary considerably, potentially resulting in varying physiological states and thereby leading to inconsistent findings. In an effort to control for caffeine, the specified duration of abstention from caffeine typically ranges from 1 to 24 h (Grant et al., 2018). Notably, protocols for abstention that correspond to the peak duration of caffeine concentrations in the bloodstream (for example, 30–120 min) are inadequate, because caffeine will be most impactful during this time (Grant et al., 2018; Nowaczewska et al., 2020). Conversely, caffeine withdrawal symptoms typically manifest ∼12 h after cessation of use, and as such, protocols requiring a duration of >12 h will also produce unfavourable outcomes. Therefore, the optimal protocol to limit the effects of caffeine appears to be ∼6 h (Grant et al., 2018).

Certainly, the duration of abstention depends largely on the time of day when the experimentation occurs. For instance, even if a protocol requires participants to refrain from consumption of caffeine for a duration of 6 h prior to testing, participants are generally less inclined to ingest caffeine in the evening and tend to postpone it until the following morning. As such, the period of abstention extends beyond the anticipated duration, probably entering a time frame of withdrawal. Abstinence for 6 h is feasible provided that the experimental setting can occur in the afternoon (e.g., the final ‘dose’ of caffeine taken at 09.00 h, with the trial commencing at 15.00 h); however, this approach would not be appropriate for research requiring overnight fasting or where diurnal variations apply (e.g., with cerebrovascular research). When morning testing is necessary, we argue that the caffeinated state is a more accurate depiction of the everyday physiological condition (at least in habitual users), and caffeine should, therefore, be allowed prior to testing, as long as the amount is minimized (<100 mg), such as one cup (250 mL) of brewed coffee (∼92 mg of caffeine) or a 30 mL espresso (∼63 mg of caffeine) (van Dam & Hu, 2022). Limiting the caffeine to <100 mg prevents participants from entering a withdrawal state without a major impact on CBF (Chen & Parrish, 2009a).

### 6.1 Other considerations

The research question at hand must be considered initially, but comparably important is the population being studied. For instance, older adults typically exhibit heightened sensitivity to the cardiovascular effects of caffeine in comparison to younger participants, as evidenced by a more pronounced increase in blood pressure (Arciero & Ormsbee, 2009). Additionally, obese individuals tend to have a diminished metabolic clearance rate and an extended half‐life of caffeine in comparison to non‐obese individuals (Starling‐Soares et al., 2023). The source of caffeine, and substances commonly present in caffeinated beverages, including l‐theanine, sucrose and taurine (Costa et al., 2023; Dodd et al., 2015), should also be considered, because it will impact the pharmacokinetics and bioavailability.

## 7 CONCLUSION

A substantial proportion of the population regularly consumes caffeine, with an average intake of ∼200 mg per day (Samoggia & Rezzaghi, 2021). Given the impracticality of exclusively recruiting participants who abstain from caffeine and acknowledging the significant prevalence of caffeine consumption, an essential question arises when conducting cerebrovascular research: what defines the ‘normal’ physiological state? In other words, does research requiring participants to abstain from caffeine accurately depict the daily physiological conditions experienced by the general population? This question is indeed complex and lacks a straightforward answer. The type and quantity of caffeine use vary considerably, but little is known about the resultant quantity and severity of withdrawal symptoms and their effects on study variables. In habitual caffeine consumers, we propose that being in a caffeinated state better represents daily physiology and has a lesser impact on research results in comparison to withdrawal, provided the caffeine intake is <100 mg within 6 h before the study.  Nevertheless, experimental protocols should be tailored to align with the primary research questions, giving thorough consideration to the potential impacts of both caffeine intake and withdrawal on the measured outcomes.

## AUTHOR CONTRIBUTIONS

Brooke R. Shepley and Anthony Richard Bain contributed to the conception of the Editorial. Each author participated in designing, writing and revising the Editorial, approved the final version and agree to be accountable for all aspects of the work in ensuring that questions related to the accuracy or integrity of any part of the work are appropriately investigated and resolved. All persons designated as authors qualify for authorship, and all those who qualify for authorship are listed.

## CONFLICT OF INTEREST

The authors declare no conflicts of interest.

## FUNDING INFORMATION

None.

## References

[eph13898-bib-0001] Addicott, M. A. , Yang, L. L. , Peiffer, A. M. , Burnett, L. R. , Burdette, J. H. , Chen, M. Y. , Hayasaka, S. , Kraft, R. A. , Maldjian, J. A. , & Laurienti, P. J. (2009). The effect of daily caffeine use on cerebral blood flow: How much caffeine can we tolerate? Human Brain Mapping, 30(10), 3102–3114. 19219847 10.1002/hbm.20732 PMC2748160

[eph13898-bib-0002] Arciero, P. J. , & Ormsbee, M. J. (2009). Relationship of blood pressure, behavioral mood state, and physical activity following caffeine ingestion in younger and older women. Applied Physiology, Nutrition, and Metabolism, 34(4), 754–762. 10.1139/H09-068 19767812

[eph13898-bib-0003] Benarroch, E. E. (2009). The locus ceruleus norepinephrine system. Neurology, 73(20), 1699–1704. 19917994 10.1212/WNL.0b013e3181c2937c

[eph13898-bib-0004] Blaha, M. , Benes, V. , Douville, C. M. , & Newell, D. W. (2007). The effect of caffeine on dilated cerebral circulation and on diagnostic CO2 reactivity testing. Journal of Clinical Neuroscience, 14(5), 464–467. 17346975 10.1016/j.jocn.2006.03.019

[eph13898-bib-0005] Bowton, D. L. , Haddon, W. S. , Prough, D. S. , Adair, N. , Alford, P. T. , & Stump, D. A. (1988). Theophylline Effect on the Cerebral Blood Flow Response to Hypoxemia. Chest, 94(2), 371–375. 3396417 10.1378/chest.94.2.371

[eph13898-bib-0006] Brassard, P. , Labrecque, L. , Smirl, J. D. , Tymko, M. M. , Caldwell, H. G. , Hoiland, R. L. , Lucas, S. J. E. , Denault, A. Y. , Couture, E. J. , & Ainslie, P. N. (2021). Losing the dogmatic view of cerebral autoregulation. Physiological Reports, 9(15), e14982. 34323023 10.14814/phy2.14982 PMC8319534

[eph13898-bib-0007] Chen, Y. , & Parrish, T. B. (2009a). Caffeine dose effect on activation‐induced BOLD and CBF responses. Neuroimage, 46(3), 577–583. 19289172 10.1016/j.neuroimage.2009.03.012 PMC2694217

[eph13898-bib-0008] Chen, Y. , & Parrish, T. B. (2009b). Caffeine's effects on cerebrovascular reactivity and coupling between cerebral blood flow and oxygen metabolism. Neuroimage, 44(3), 647–652. 19000770 10.1016/j.neuroimage.2008.09.057 PMC2654762

[eph13898-bib-0009] Costa, R. , Rocha, C. , & Santos, H. (2023). Cardiovascular and cerebrovascular response to Redbull^®^ energy drink intake in young adults. Anatolian Journal of Cardiology, 27(1), 19–25. 36680443 10.14744/AnatolJCardiol.2022.2315 PMC9893714

[eph13898-bib-0010] Couturier, E. , Laman, D. , van Duijn, M. , & van Duijn, H. (1997). Influence of caffeine and caffeine withdrawal on headache and cerebral blood flow velocities. Cephalalgia, 17(3), 188–190. 9170342 10.1046/j.1468-2982.1997.1703188.x

[eph13898-bib-0011] Dews, P. B. , O'Brien, C. P. , & Bergman, J. (2002). Caffeine: Behavioral effects of withdrawal and related issues. Food and Chemical Toxicology, 40(9), 1257–1261. 12204389 10.1016/s0278-6915(02)00095-9

[eph13898-bib-0012] Dodd, F. L. , Kennedy, D. O. , Riby, L. M. , & Haskell‐Ramsay, C. F. (2015). A double‐blind, placebo‐controlled study evaluating the effects of caffeine and L‐theanine both alone and in combination on cerebral blood flow, cognition and mood. Psychopharmacology, 232(14), 2563–2576. 25761837 10.1007/s00213-015-3895-0 PMC4480845

[eph13898-bib-0013] D'Urzo, A. D. , Jhirad, R. , Jenne, H. , Avendano, M. A. , Rubinstein, I. , D'Costa, M. , Goldstein, R. S. , & Rubenstein, I. (1990). Effect of caffeine on ventilatory responses to hypercapnia, hypoxia, and exercise in humans. Journal of Applied Physiology, 68(1), 322–328. 2312473 10.1152/jappl.1990.68.1.322

[eph13898-bib-0014] Fredholm, B. B. , Bättig, K. , Holmén, J. , Nehlig, A. , & Zvartau, E. E. (1999). Actions of caffeine in the brain with special reference to factors that contribute to its widespread use. Pharmacological Reviews, 51(1), 83–133. 10049999

[eph13898-bib-0015] Gil, Y.‐E. , Lee, M. J. , Cho, S. , & Chung, C.‐S. (2022). Effect of caffeine and caffeine cessation on cerebrovascular reactivity in patients with migraine. Headache, 62(2), 169–175. 35114026 10.1111/head.14263

[eph13898-bib-0016] Grant, S. S. , Magruder, K. P. , & Friedman, B. H. (2018). Controlling for caffeine in cardiovascular research: A critical review. International Journal of Psychophysiology, 133, 193–201. 29981767 10.1016/j.ijpsycho.2018.07.001

[eph13898-bib-0017] Hamner, J. W. , & Tan, C. O. (2014). Relative contributions of sympathetic, cholinergic, and myogenic mechanisms to cerebral autoregulation. Stroke, 45(6), 1771–1777. 24723314 10.1161/STROKEAHA.114.005293 PMC4102642

[eph13898-bib-0018] Hoiland, R. L. , Bain, A. R. , Tymko, M. M. , Rieger, M. G. , Howe, C. A. , Willie, C. K. , Hansen, A. B. , Flück, D. , Wildfong, K. W. , Stembridge, M. , Subedi, P. , Anholm, J. , & Ainslie, P. N. (2017). Adenosine receptor‐dependent signaling is not obligatory for normobaric and hypobaric hypoxia‐induced cerebral vasodilation in humans. Journal of Applied Physiology (Bethesda, Md.: 1985), 122(4), 795–808. 28082335 10.1152/japplphysiol.00840.2016 PMC5407198

[eph13898-bib-0019] Hoiland, R. L. , Fisher, J. A. , & Ainslie, P. N. (2019). Regulation of the cerebral circulation by arterial carbon dioxide. In Comprehensive Physiology (pp. 1101–1154). John Wiley & Sons, Ltd. 10.1002/cphy.c180021 31187899

[eph13898-bib-0020] Johansson, B. , Ahlberg, S. , van der Ploeg, I. , Brené, S. , Lindefors, N. , Persson, H. , & Fredholm, B. B. (1993). Effect of long term caffeine treatment on A1 and A2 adenosine receptor binding and on mRNA levels in rat brain. Naunyn‐Schmiedeberg's Archives of Pharmacology, 347(4), 407–414. 8510768 10.1007/BF00165391

[eph13898-bib-0021] Jones, H. E. , Herning, R. I. , Cadet, J. L. , & Griffiths, R. R. (2000). Caffeine withdrawal increases cerebral blood flow velocity and alters quantitative electroencephalography (EEG) activity. Psychopharmacology, 147(4), 371–377. 10672630 10.1007/s002130050005

[eph13898-bib-0022] Juliano, L. M. , & Griffiths, R. R. (2004). A critical review of caffeine withdrawal: Empirical validation of symptoms and signs, incidence, severity, and associated features. Psychopharmacology, 176(1), 1–29. 15448977 10.1007/s00213-004-2000-x

[eph13898-bib-0023] Juliano, L. M. , Kardel, P. G. , Harrell, P. T. , Muench, C. , & Edwards, K. C. (2019). Investigating the role of expectancy in caffeine withdrawal using the balanced placebo design. Human Psychopharmacology: Clinical and Experimental, 34(2), e2692. 30861208 10.1002/hup.2692

[eph13898-bib-0024] Kennedy, D. O. , & Haskell, C. F. (2011). Cerebral blood flow and behavioural effects of caffeine in habitual and non‐habitual consumers of caffeine: A near infrared spectroscopy study. Biological Psychology, 86(3), 298–306. 21262317 10.1016/j.biopsycho.2010.12.010

[eph13898-bib-0025] Lane, J. D. (1997). Effects of brief caffeinated‐beverage deprivation on mood, symptoms, and psychomotor performance. Pharmacology Biochemistry and Behavior, 58(1), 203–208. 9264092 10.1016/s0091-3057(97)00007-5

[eph13898-bib-0026] Mesas, A. E. , Leon‐Muñoz, L. M. , Rodriguez‐Artalejo, F. , & Lopez‐Garcia, E. (2011). The effect of coffee on blood pressure and cardiovascular disease in hypertensive individuals: A systematic review and meta‐analysis123. American Journal of Clinical Nutrition, 94(4), 1113–1126. 21880846 10.3945/ajcn.111.016667

[eph13898-bib-0027] Nam, H. W. , McIver, S. R. , Hinton, D. J. , Thakkar, M. M. , Sari, Y. , Parkinson, F. E. , Haydon, P. G. , & Choi, D.‐S. (2012). Adenosine and glutamate signaling in neuron‐glial interactions: Implications in alcoholism and sleep disorders. Alcoholism, Clinical and Experimental Research, 36(7), 1117–1125. 22309182 10.1111/j.1530-0277.2011.01722.x PMC3349794

[eph13898-bib-0028] Nowaczewska, M. , Wiciński, M. , & Kaźmierczak, W. (2020). The ambiguous role of caffeine in migraine headache: From trigger to treatment. Nutrients, 12(8), 2259. 32731623 10.3390/nu12082259 PMC7468766

[eph13898-bib-0029] Papadelis, C. , Kourtidou‐Papadeli, C. , Vlachogiannis, E. , Skepastianos, P. , Bamidis, P. , Maglaveras, N. , & Pappas, K. (2003). Effects of mental workload and caffeine on catecholamines and blood pressure compared to performance variations. Brain and Cognition, 51(1), 143–154. 12633594 10.1016/s0278-2626(02)00530-4

[eph13898-bib-0030] Pelligrino, D. A. , Xu, H.‐L. , & Vetri, F. (2010). Caffeine and the control of cerebral hemodynamics. Journal of Alzheimer's Disease : JAD, 20(Suppl 1), S51–S62. 10.3233/JAD-2010-091261 PMC2944660 20182032

[eph13898-bib-0031] Phillips‐Bute, B. G. , & Lane, J. D. (1997). Caffeine withdrawal symptoms following brief caffeine deprivation. Physiology & Behavior, 63(1), 35–39. 9402612 10.1016/s0031-9384(97)00384-3

[eph13898-bib-0032] Pincomb, G. A. , Lovallo, W. R. , Passey, R. B. , & Wilson, M. F. (1988). Effect of behavior state on caffeine's ability to alter blood pressure. American Journal of Cardiology, 61(10), 798–802. 3354444 10.1016/0002-9149(88)91069-7

[eph13898-bib-0033] Powers, S. K. , Dodd, S. , Woodyard, J. , & Mangum, M. (1986). Caffeine alters ventilatory and gas exchange kinetics during exercise. Medicine & Science in Sports & Exercise, 18(1), 101. 3959852

[eph13898-bib-0034] Ribeiro, J. A. , & Sebastião, A. M. (2010). Caffeine and adenosine. Journal of Alzheimer's Disease, 20(s1), S3–S15. 10.3233/JAD-2010-1379 20164566

[eph13898-bib-0035] Richmond, G. H. (1949). Action of caffeine and aminophylline as respiratory stimulants in man. Journal of Applied Physiology, 2(1), 16–23. 18133123 10.1152/jappl.1949.2.1.16

[eph13898-bib-0036] Samoggia, A. , & Rezzaghi, T. (2021). The consumption of caffeine‐containing products to enhance sports performance: An application of an extended model of the theory of planned behavior. Nutrients, 13(2), 344. 33498924 10.3390/nu13020344 PMC7912121

[eph13898-bib-0037] Shi, D. , & Daly, J. W. (1999). Chronic effects of xanthines on levels of central receptors in mice. Cellular and Molecular Neurobiology, 19(6), 719–732. 10456233 10.1023/A:1006901005925 PMC11545639

[eph13898-bib-0038] Starling‐Soares, B. , Pereira, M. , & Renke, G. (2023). Extrapolating the coffee and caffeine (1,3,7‐trimethylxanthine) effects on exercise and metabolism—A concise review. Nutrients, 15(24), 5031. 38140290 10.3390/nu15245031 PMC10745355

[eph13898-bib-0039] Turnbull, D. , Rodricks, J. V. , Mariano, G. F. , & Chowdhury, F. (2017). Caffeine and cardiovascular health. Regulatory Toxicology and Pharmacology, 89, 165–185. 28756014 10.1016/j.yrtph.2017.07.025

[eph13898-bib-0040] Umemura, T. , Ueda, K. , Nishioka, K. , Hidaka, T. , Takemoto, H. , Nakamura, S. , Jitsuiki, D. , Soga, J. , Goto, C. , Chayama, K. , Yoshizumi, M. , & Higashi, Y. (2006). Effects of acute administration of caffeine on vascular function. American Journal of Cardiology, 98(11), 1538–1541. 17126666 10.1016/j.amjcard.2006.06.058

[eph13898-bib-0041] van Dam, R. M. , & Hu, F. B. (2022). Caffeine consumption and cardiovascular health. Nature Reviews Cardiology, 19(7), 429–430. 10.1038/s41569-022-00719-4 35581338

[eph13898-bib-0042] Willie, C. K. , Tzeng, Y.‐C. , Fisher, J. A. , & Ainslie, P. N. (2014). Integrative regulation of human brain blood flow. The Journal of Physiology, 592(5), 841–859. 24396059 10.1113/jphysiol.2013.268953 PMC3948549

[eph13898-bib-0043] Xu, F. , Liu, P. , Pekar, J. J. , & Lu, H. (2015). Does acute caffeine ingestion alter brain metabolism in young adults? Neuroimage, 110, 39–47. 25644657 10.1016/j.neuroimage.2015.01.046 PMC4380776

[eph13898-bib-0044] Zhu, W. M. , Neuhaus, A. , Beard, D. J. , Sutherland, B. A. , & DeLuca, G. C. (2022). Neurovascular coupling mechanisms in health and neurovascular uncoupling in Alzheimer's disease. Brain, 145(7), 2276–2292. 35551356 10.1093/brain/awac174 PMC9337814

